# Parkinsonism Secondary to Hydrocephalus Caused by Neurocysticercosis

**DOI:** 10.7759/cureus.7887

**Published:** 2020-04-29

**Authors:** Sebastião Carlos de Sousa Oliveira, Dina Andressa Martins Monteiro, Giselle Furtado Silva, Lucas Tadeu Rocha Santos, Espártaco Moraes Lima Ribeiro

**Affiliations:** 1 Neurological Surgery, Federal University of Ceara, Sobral, BRA; 2 Neurology, Federal University of Ceara, Sobral, BRA; 3 Internal Medicine, Federal University of Ceara, Sobral, BRA

**Keywords:** neurocysticercosis, hydrocephalus, parkinsonism

## Abstract

The diagnosis of parkinsonism is established by the presence of tremor, stiffness and bradykinesia alongside with neurological examination, requiring the exclusion of secondary causes such as stroke, hydrocephalus and infectious diseases. Included in this last category, neurocysticercosis is a disease caused by Taenia solium, with a variable clinical presentation that can include epileptic seizures, hydrocephalus and rarely parkinsonism. In the reported case, the syndrome is a consequence of lesions in the nigrostriatal dopaminergic pathway caused by the implant and mass effect of the cysticercus. The authors report a case of parkinsonism in a 59-year-old woman with a previous history of neurocysticercosis who presented with hydrocephalus on magnetic resonance imaging exam. The patient was treated with pharmacological therapy and ventriculoperitoneal shunt, progressing with amelioration of the symptoms presented.

## Introduction

Parkinsonism is characterized by the triad of tremor, stiffness and bradykinesia [1]. Medical history and neurological examination are responsible for the diagnosis of parkinsonian syndromes, and tests such as MRI have the role of excluding underlying disease, such as stroke and normal pressure hydrocephalus. The differential diagnosis is much broader and includes neurodegenerative, metabolic, neoplastic, drug induced parkinsonism and infectious diseases, including neurocysticercosis (NC).

Cysticercosis is a disease caused by Taenia solium larva and has two syndromic presentations: extraneural and NC. The latter, in endemic countries, is considered the main cause of seizures in adults [2]. Other presentations of NC are focal neurological deficits, hydrocephalus, meningismus, arachnoiditis and some movement disorders, such as tremor, chorea, dystonia and parkinsonism.

## Case presentation

**Figure 1:**
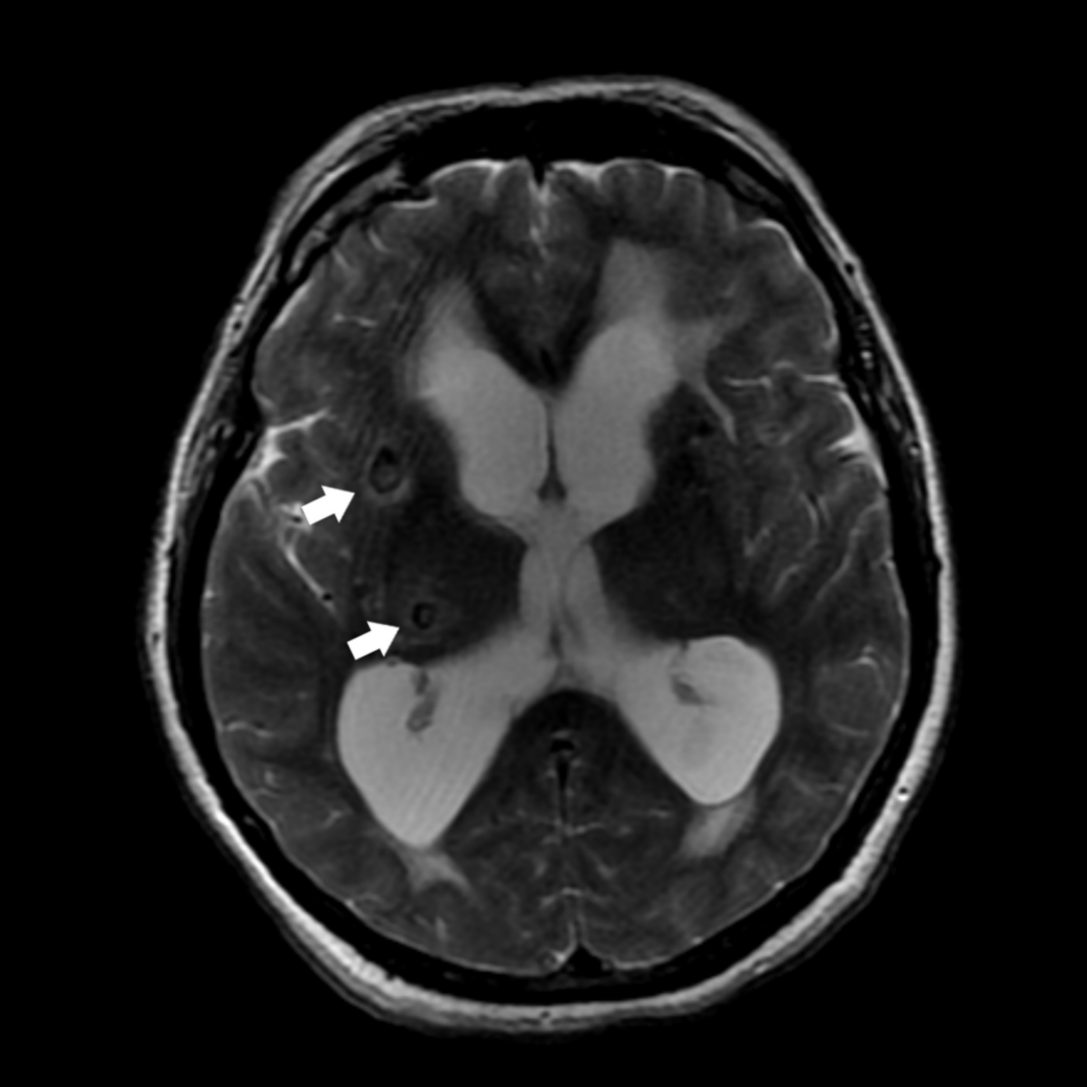
MRI T2-weighted axial. T2-weighted axial MRI image showing subcortical cystic lesions with perilesional hyperintensity and presence of scolex (arrows).

**Figure 2:**
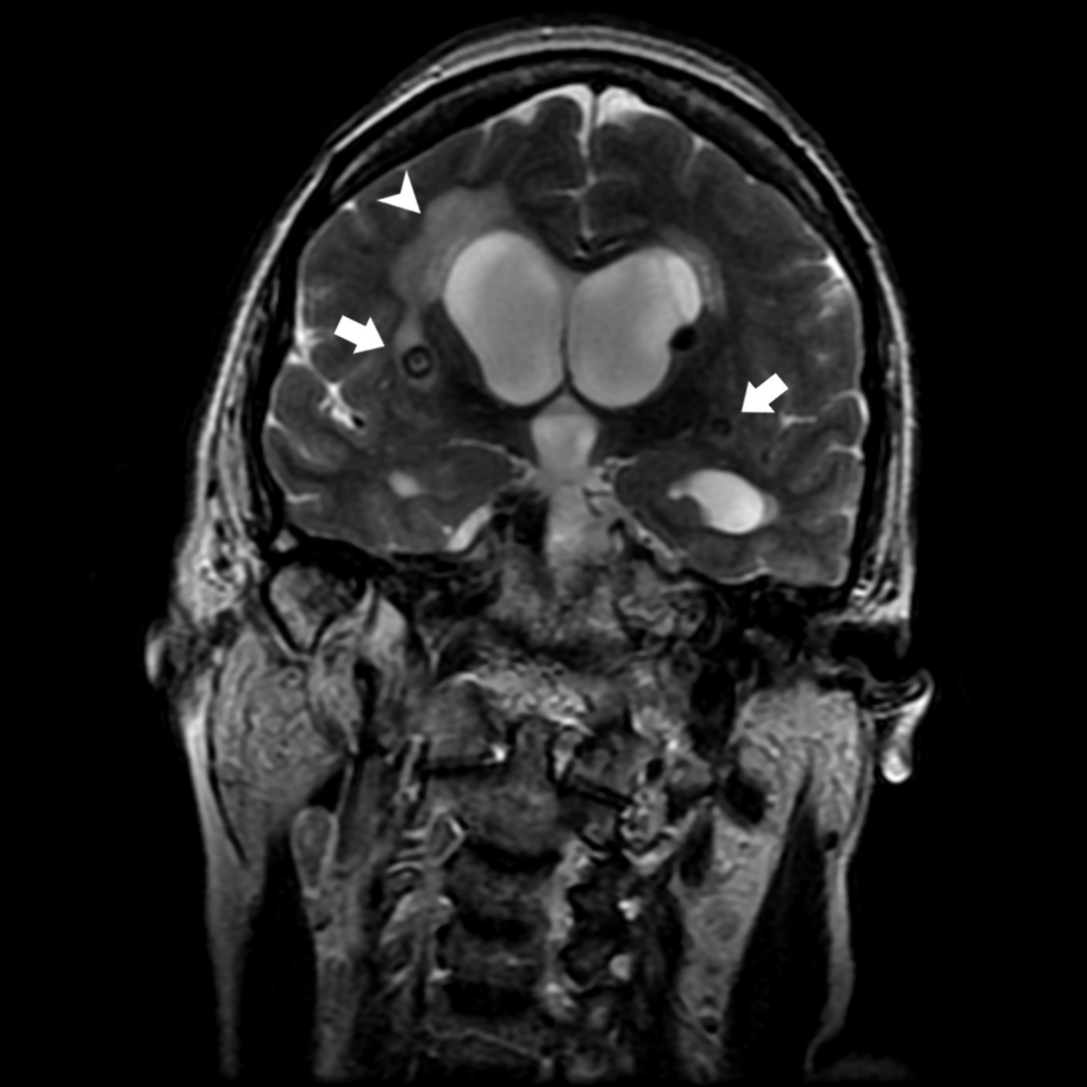
MRI T2-weighted coronal. T2-weighted coronal MRI image showing cysticerci in brain parenchyma (left arrow) and subarachnoid space (right arrow)  and signs of transependymal edema (arrowhead).

A 59-year-old woman was admitted with progressive symptoms of urinary incontinence, amnesia for recent events, failure to recognize family members and difficulty walking since four months ago. She had a wide-based gait and stiffness predominating in the lower limbs, which later evolved to incapacity to walk and bradykinesia. The patient was diagnosed with NC 20 years ago and epilepsy approximately 15 years ago, treated with phenobarbital. She sought assistance from a neurologist, who performed a lumbar puncture in order to assess the persistence of symptoms after removing excess of cerebrospinal fluid (CSF). The lumbar puncture showed CSF of normal characteristics. The patient showed no improvement in symptoms after the procedure and was referred to the hospital for diagnostic investigation. Admission laboratory tests showed no pathological changes. Axial (Figure 1) and coronal (Figure 2) sections of a T2-weighted MRI of the brain revealed cystic lesions in the subcortical region with the presence of scolex associated with perilesional hyperintensity, suggestive of NC in vesicular stage associated with hydrocephalus and signals of transependymal resorption.

**Figure 3:**
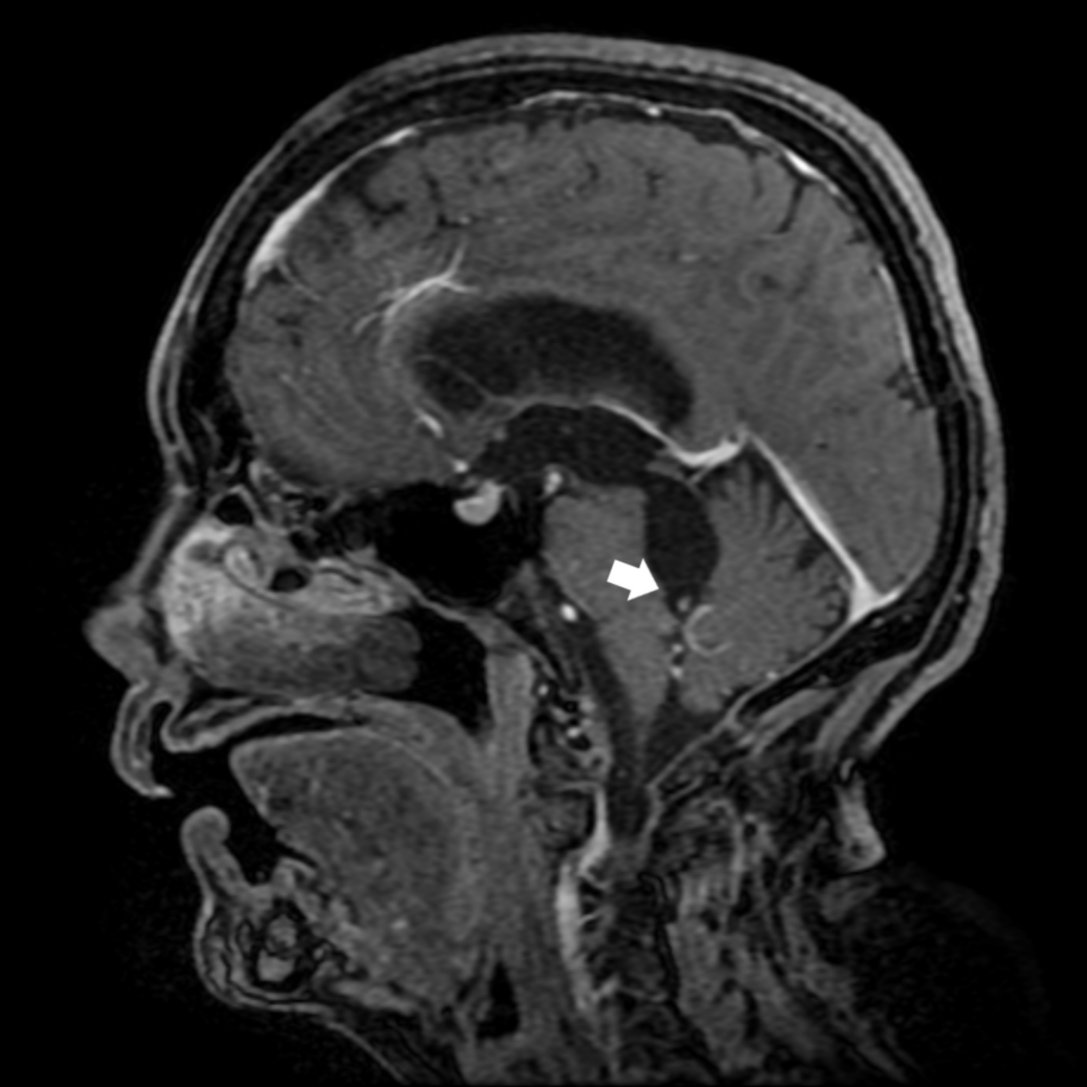
MRI T1-weighted sagittal. T1-weighted sagittal MRI image showing the presence of cysticerci causing obstruction of the Magendie's foramen.

**Figure 4:**
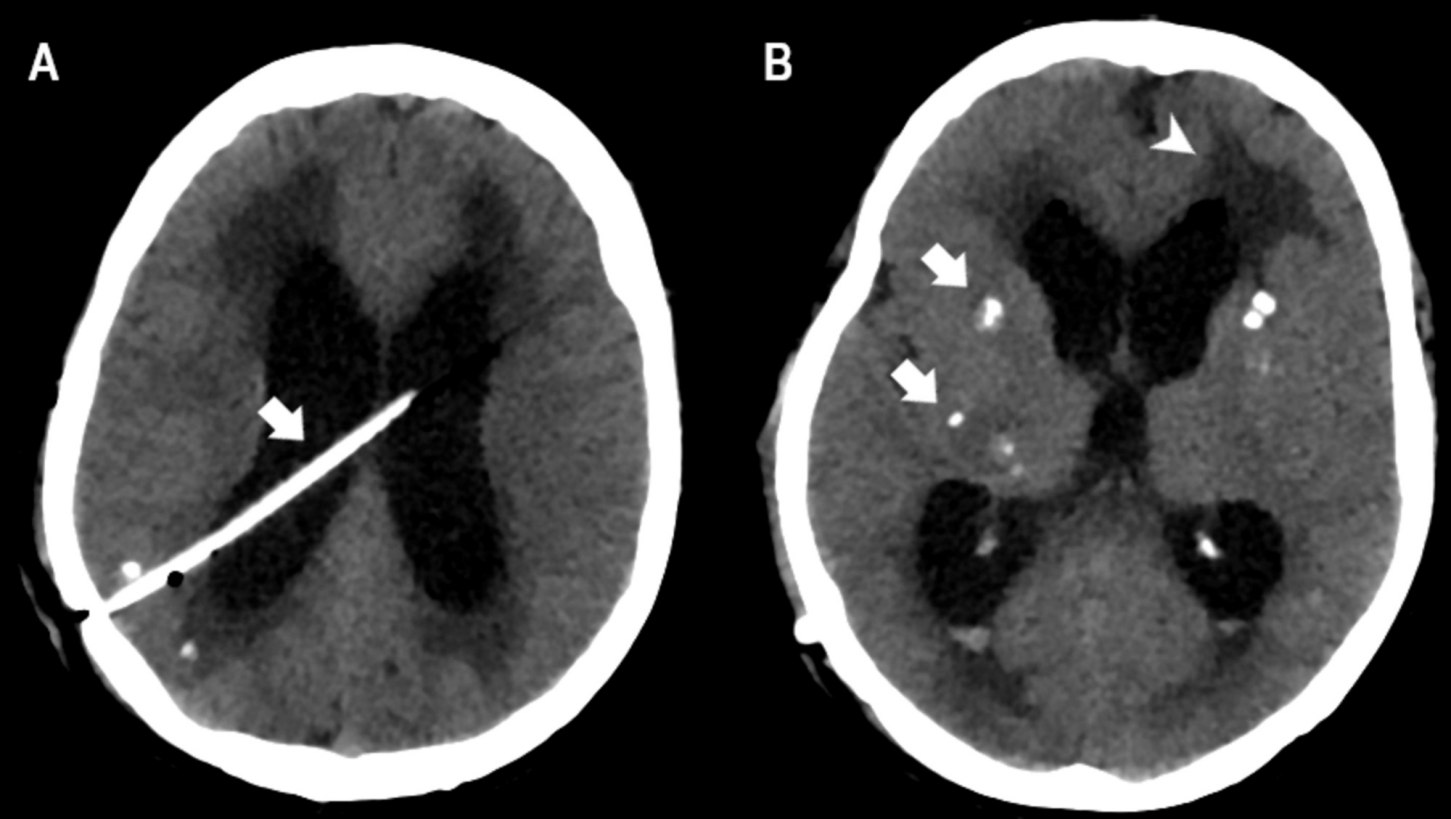
Noncontrast-enhanced cranial CT (A) Noncontrast-enhanced cranial CT showing a well-placed ventricular drain (arrow). (B) Cranial CT showing multiple subcortical calcifications (arrows) associated with transependymal resorption (arrowhead).

On T1-weighted sagittal section, the presence of a cysticercus in the foramen of Magendie was noted (Figure 3). The patient underwent ventriculoperitoneal shunt (VPS) insertion, and evolved with clinical improvement of the symptoms. A noncontrast-enhanced cranial CT (Figure 4) was performed to assess the postoperative status, which showed multiple cortical and subcortical calcifications and well-placed ventricular drain. The patient was discharged using albendazole and praziquantel for 14 days, on an outpatient basis. 

## Discussion

NC is a parasitosis of the central nervous system caused by the helminth Taenia solium. This parasite has a life cycle in which man represents the only definitive host and the pig represents the intermediate host. Central nervous system infection in humans occurs when the individual accidentally ingests Taenia solium eggs, or when the larval stage has an affinity for the central nervous system tissue, causing NC [3]. NC can present several clinical manifestations according to the type, quantity and location of cysticerci in the nervous system. Common clinical manifestations of NC include epileptic seizures, cysticercotic meningitis, dementia, obstructive hydrocephalus and, more rarely, parkinsonism [3,4].

Parkinsonism, although rare, can be caused by lesions in the nigrostriatal dopaminergic pathway through tissue destruction. This destruction can be caused by direct implants of cysticerci in the tissue of the nigrostriatal pathway or by a mass effect resulting from the implantation of cysticerci in surrounding brain areas. The implantation of cysticerci in the IV ventricle or in the cisterna magna can prevent the flow of cerebrospinal fluid, resulting in hydrocephalus, which in turn can cause mass effect in the nigrostriatal pathway. Local edema resulting from the host's inflammatory reaction to implantation of cysticerci can also cause an increase in intracranial pressure and, therefore, generate or worsen lesions due to a mass effect in the nigrostriatal pathway [5]. 

The clinical presentation of NC is directly related to the location of the lesion. The disease in its intraparenchymal form, which accounts for 60% of the cases, is related to a convulsive condition due to the inflammatory response associated with degeneration or calcification of the cysticercus. Regarding the intraventricular form, which represents a total of 10% to 20% of the cases, it can cause obstructive hydrocephalus [2]. In the case described, both types of lesions are observed and may explain the parkinsonian syndrome. In a retrospective study, of the 590 patients studied, 23 (3.8%) developed movement disorders, in which 15 (65%) had parkinsonism [4]. Unlike what was observed in the case described, the time to onset of motor symptoms varied up to 140 days after diagnosis of NC. However, in other studies, this interval can vary up to 30 years, generally occurring between three and five years after infection [6]. In this group of patients, the clinical presentation of parkinsonism occurs differently, with bilateral involvement, axial hypokinesia, mild stiffness, infrequent resting tremor, instability, constant falls and frontal gait disorder. The exclusion of differential diagnoses is essential.

In the case presented, differential diagnoses were normal pressure hydrocephalus and Parkinson's disease. The first is marked by the triad of dementia, gait changes and urinary incontinence, being excluded as a diagnostic hypothesis due to the lack of improvement in symptoms after lumbar puncture, the subacute evolution and the presence of obstructive hydrocephalus [1]. Parkinson's disease was disregarded for the clinical case due to improvement of the symptoms with the treatment of the hydrocephalus and NC.

The diagnosis of NC can be made with the presence of one of the three absolute criteria, which are histopathological examination showing the parasite in the lesion, image examination showing scolex or direct visualization of the parasite in the fundus examination. In addition to the absolute criteria, there are two other categories of diagnostic criteria: neuroimaging criteria (Table 1) and clinical criteria (Table 2) [7].

**Table 1:** Neuroimaging criteria for the diagnosis of neurocysticercosis [7].

Major neuroimaging criteria	Confirmative neuroimaging criteria	Minor neuroimaging criteria
Cystic lesions without a discernible scolex	Resolution of cystic lesions after cysticidal drug therapy	Obstructive hydrocephalus (symmetric or asymmetric) or abnormal enhancement of basal leptomeninges
Enhancing lesions	Spontaneous resolution of single small enhancing lesions	
Multilobulated cystic lesions in the subarachnoid space	Migration of ventricular cysts documented on sequential neuroimaging studies	

**Table 2:** Clinical criteria for the diagnosis of neurocysticercosis [7].

Major clinical/exposure	Minor clinical/exposure
Detection of specific anticysticercal antibodies or cysticercal antigens by well-standardized immunodiagnostic tests	Obstructive hydrocephalus (symmetric or asymmetric) or abnormal enhancement of basal leptomeninges
Cysticercosis outside the central nervous system	
Evidence of a household contact with Taenia solium infection	

**Figure 5:**
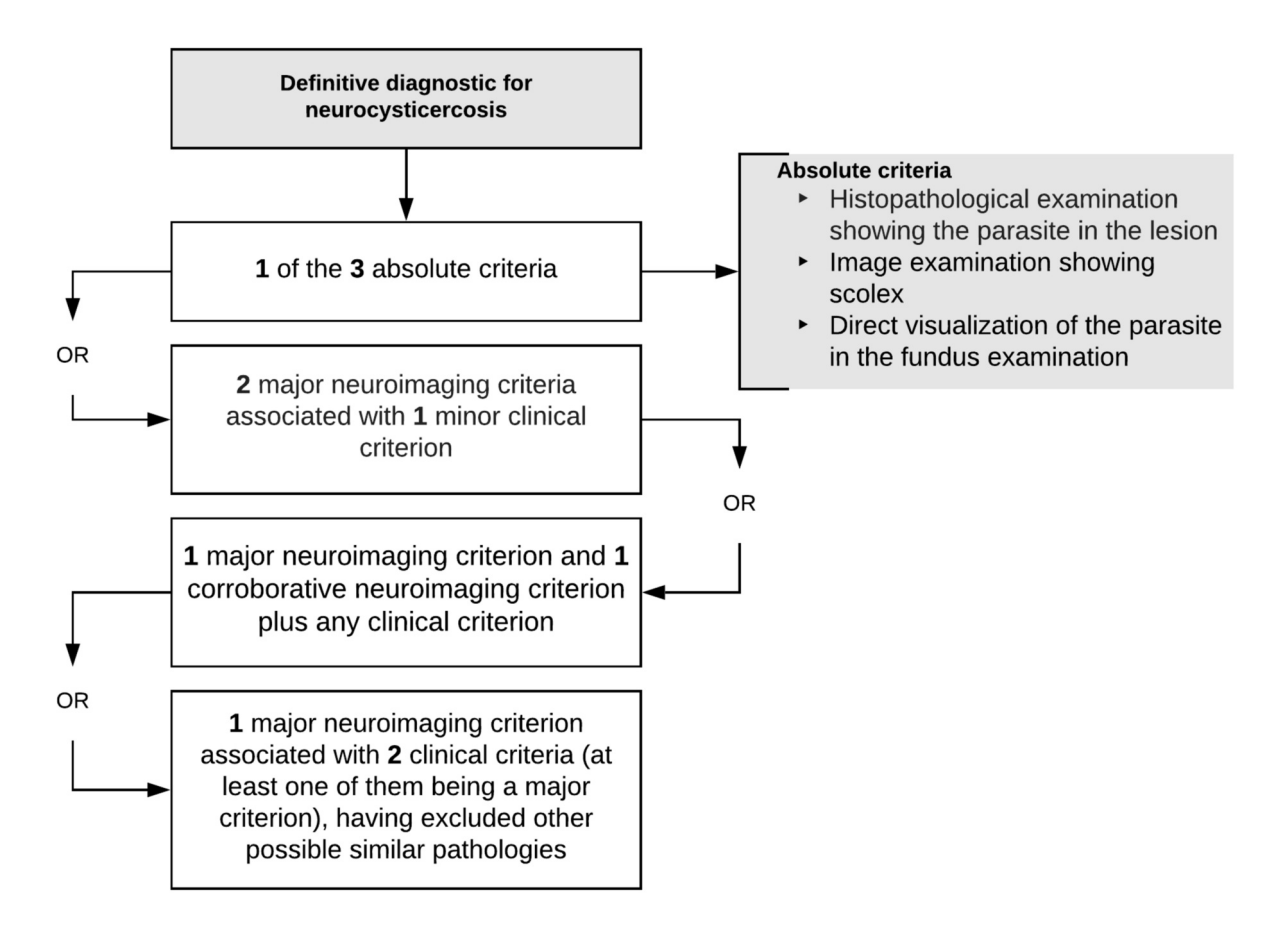
Definitive diagnostic for neurocysticercosis.

The diagnosis of NC can be definitively established with the presence of two major neuroimaging criteria associated with a minor clinical criterion. The addition of a major neuroimaging criterion, a corroborative neuroimaging criterion and any clinical criterion also establishes the definitive diagnosis of the disease. In addition, the definitive diagnosis can also be established with the presence of a major neuroimaging criterion associated with two clinical criteria (at least one of them being a major criterion), having excluded other possible similar pathologies (Figure 5) [7].

As for the clinical case presented in this article, the treatment for parkinsonian syndrome presented by the patient is mainly the treatment of the underlying etiology (NC and hydrocephalus), obtaining improvement with the insertion of a VPS. In cases of active cystic lesions, a recent study showed that the combined therapy of praziquantel 50 mg/kg/day and albendazole 15 mg/kg/day for 10 days was more effective than the use of a single drug, without causing greater side effects. At the beginning of treatment, corticosteroids are often used for a few days associated with cysticide therapy to minimize cerebral edema caused by the death of cysticerci. Dexamethasone and prednisone are the most frequently used medications [8].

The process of resolving acute infection can lead to the formation of granulomas, which can remain to cause the symptoms of the disease in the patient during this process. It is possible that calcified residues remain in the nervous system, which can generate clinical manifestations for a prolonged period of time. Therefore, the use of medications directed to these symptoms may be necessary. Due to the diversity of possible clinical manifestations of the disease, the treatment of NC is a very comprehensive topic, which can encompass several other specific therapeutic strategies, such as VPS and the use of levodopa [8].

## Conclusions

NC is a disease that presents itself clinically in several ways, one of which is parkinsonism. Although rare, parkinsonism secondary to NC can cause numerous losses to the patient's life. Therefore, it is extremely relevant that the medical community knows how to recognize this condition, so that it is possible to establish an appropriate treatment for the patient and improve the individual's quality of life. However, it should be emphasized that additional investigation on the subject is needed in order to establish diagnoses and treatments with greater security.
